# Specific antibody responses against membrane proteins of erythrocytes infected by *Plasmodium falciparum *of individuals briefly exposed to malaria

**DOI:** 10.1186/1475-2875-9-276

**Published:** 2010-10-11

**Authors:** Albin Fontaine, Matthieu Pophillat, Stéphanie Bourdon, Claude Villard, Maya Belghazi, Patrick Fourquet, Claude Durand, Didier Lefranc, Christophe Rogier, Thierry Fusai, Lionel Almeras

**Affiliations:** 1 Unité de recherche en biologie et épidémiologie parasitaires (URBEP) - UMR6236 - IFR48, Antenne Marseille de l'Institut de Recherche Biomédicale des Armées (IRBA), BP 60109, 13 262 Marseille Cedex 07, France; 2 Centre d'Immunologie de Marseille Luminy (CIML), Institut National de la Santé et de la Recherche Médicale, Centre National de la Recherche Scientifique, Université de la Méditerranée, Parc Scientifique de Luminy, Case 906, 13288 Marseille Cedex 09, Marseille, France; 3 Plateforme protéomique MapTimone, INSERM UMR 911 CRO2, Faculté de Pharmacie/Université de la Méditerranée, 27 Bd Jean Moulin, 13385 Marseille Cedex 05, France; 4 Centre d'Analyse Protéomique de Marseille (CAPM), Institut Jean Roche, Faculté de médecine Nord, Marseille, France; 5 Laboratoire d'immunologie EA2686, Faculté de Médecine, 1, Place de Verdun, 59045 Lille Cedex, France

## Abstract

**Background:**

*Plasmodium falciparum *infections could lead to severe malaria, principally in non-immune individuals as children and travellers from countries exempted of malaria. Severe malaria is often associated with the sequestration of *P. falciparum*-infected erythrocytes in deep micro-vascular beds via interactions between host endothelial receptors and parasite ligands expressed on the surface of the infected erythrocyte. Although, serological responses from individuals living in endemic areas against proteins expressed at surface of the infected erythrocyte have been largely studied, seldom data are available about the specific targets of antibody response from travellers.

**Methods:**

In order to characterize antigens recognized by traveller sera, a comparison of IgG immune response against membrane protein extracts from uninfected and *P. falciparum*-infected red blood cells (iRBC), using immunoblots, was performed between non exposed individuals (*n *= *31*) and briefly exposed individuals (BEI) *(n = 38*) to malaria transmission.

**Results:**

Immune profile analysis indicated that eight protein bands from iRBC were significantly detected more frequently in the BEI group. Some of these antigenic proteins were identified by an original immuno-proteomic approach.

**Conclusion:**

Collectively, these data may be useful to characterize the singular serological immune response against a primary malaria infection in individuals briefly exposed to transmission.

## Background

The protozoan parasite *Plasmodium falciparum *is the causative agent of the most virulent form of human malaria, affecting about 500 million of persons yearly and leading to nearly two million deaths, mainly in Africa [1]. Individuals residing in endemic areas of parasite transmission acquired gradually a protective immunity to *P. falciparum *malaria after numerous disease episodes during childhood. This immunity is not sterilizing but is protective against clinical disease and especially severe malaria [2,3]. Children from endemic areas and travellers from non-endemic countries, considered as non-immune individuals, are particularly at risk of dying from severe malaria [4].

Blood stages of *P. falciparum *are responsible for all the clinical symptoms of malaria including severe cases such as severe anaemia or visceral disorders [5]. These visceral disorders are associated with sequestration of infected red blood cells (iRBC) [6,7]. This sequestration phenomenon is due to interactions between endothelial receptors and parasite proteins expressed at the surface of iRBC. The abundance and the long time display of the blood-stage malaria parasites in the human host render the proteins from the erythrocytic parasite stages an important target for the immune system. The absence of antigen processing in erythrocytes prevents the iRBC destruction by specific MHC-restricted T-cell response. Immunity to blood stage malaria parasites is thus primarily conferred by humoral immune responses [8]. Additionally, the passive transfer of IgG from highly exposed individuals (HEI) to non-immune patients could confer a protective effect to clinical symptoms of malaria [8-10]. Among proteins supposed to induce a protective immunity, the surface-expressed *P. falciparum *erythrocyte protein 1 (PfEMP-1) have been largely studied [11-13]. However, others erythrocytic parasite stage proteins were reported to elicit an immune response [14-17]. Collectively, these data suggest that analysis of the serological immune response from exposed individuals to malaria (briefly or continuously) could be informative for the understanding of the protective immune response development.

Serological responses from individuals living in hyperendemic areas for malaria have been largely studied. Several blood stage antigens have been characterized, and some of them are candidates for vaccine trials (for review [18,19]). Although some studies have assessed the antibody response to pre-erythrocytic antigens in travellers [20-22], seldom data are available concerning the antibody response against blood stage antigens from non-immune healthy adults briefly exposed to malaria transmission, such as travellers or individuals living in area where malaria is under elimination.

The aim of this study was to identify iRBC membrane antigenic protein repertoire recognized specifically by briefly exposed individuals (BEI). An immunoblot approach allowed us to define a singular BEI IgG response against membrane protein extract from iRBC compared to non-exposed individuals (NEI). This specific serological immune response could be useful to estimate individual exposure to malaria transmission, and to understand the first stages of the immune responses to primary malaria infection.

## Methods

### Population studied

Five French soldier companies (*n *= *751*, mean age ± SD: 25.3 ± 4.8 years), who travelled during a five-month period in tropical Africa (Gabon or Ivory Coast, from 2002 to 2007), were included in this study. Individuals used mandatory anti-malaria prophylaxis including anti-vectorial equipment, such as permethrin-impregnated bed nets, repellents and long-sleeve battle dress at night and chemoprophylaxis (100 mg of doxycycline per day). Blood samples were collected one week after the return to France and sera were stored at -80°C. Sera from individuals living in France (*n *= *31*, mean age ± SD: 31.2 ± 6.5 years, Caucasians), who have never been exposed to malaria vectors and parasite were used as negative controls. Sera from highly exposed individuals (HEI) to malaria from Congo (*n *= *9*), gratefully provided by Pr. Hovette (Centre médical de secours Total, Pointe Noire, Republic of the Congo) and from Senegal (*n *= *5*), were used as positive controls. All participants gave their written informed consent to take part in the study and Marseille-2 University ethical committee has approved the protocol (Ethics Statement).

### Parasite culture

The RP8 *P. falciparum *cytoadherent strain was selected from the Palo Alto (FUP)1 strain by panning on Human Umbilical Vein Endothelial Cells (HUVEC, ATCC number: PCS-100-010), as described by Fried and Duffy [23]. This cell line express CD36 and ICAM-1 which are well known host receptors involved in cytoadherence of iRBC. Parasites were maintained in continuous culture in candle jar as previously described [24,25]. Briefly, parasites were cultured at 5% haematocrit of type O^+ ^human RBC suspended in RPMI 1640 (Invitrogen, Paisley, UK) supplemented with 25 mM HEPES (Invitrogen), 6 mM D-glucose (Sigma-Aldrich, St Louis, USA), 23 mM NaHCO_3 _(Invitrogen), 32 μg/ml neomycine (Sigma-Aldrich), 0.2 mM L-glutamine (Invitrogen), 0.25 μg/ml orotic acid (Sigma-Aldrich), 0.5 μg/ml hypoxanthine (Sigma-Aldrich) and 10% of heat-inactivated type O^+ ^human serum, at 37°C in a gas mixture of 3% CO_2_, 17% O_2_. Parasitaemia and erythrocytic cycle stages proportion were monitored daily microscopically by examination of blood smear stained with Giemsa-stained (RAL^® ^555, France).

### Liquid indirect immunofluorescence assay (L-IFA)

RBC and iRBC from *in vitro *cultures with about 10% parasitaemia were used to perform L-IFA as previously described [26,27]. Briefly, 50 μl of cell suspension were washed two times in RPMI 1640 at room temperature and incubated in DAPI (40 μg/ml) (Invitrogen, USA) for 30 min at 37°C. After 3 washing in PBS (Invitrogen), immuno-staining was started by incubating cells with 50 μl of diluted sera for 30 min on ice. Each sera was individually analysed using dilutions of 1:40, 1:80, 1:160, 1:320, 1:640 and 1:1,280 in PBS pH 7.4. Positive and negative control sera were used at 1:40 dilution. After two washes with RPMI 1640, alexa-fluor 488 goat anti-human immunoglobulin G (H+L) (Molecular Probes, USA) was added to the cell suspension and incubated for 30 min on ice. A droplet of cell suspension was laid on a microscopy slide, and antibody binding and DNA staining were assessed by fluorescence microscopy on a Nikon eclipse E800 fluorescence microscope (100 × magnification). Sample titres were determined by the maximum dilution where the fluorescence could be detected.

### Sample preparation

*Plasmodium falciparum *adherent mature stages (trophozoite and schizonte stages) were enriched by Plasmion flotation (Fresenius France Pharma, Louvier, France) as described elsewhere [25,28]. Infected erythrocytes were washed two times in PBS medium and lysed in cold hypotonic medium (H_2_O-saponin 0.1%, Sigma) for 2 min. Free parasites were discarded by centrifugation (9 300 × g for 4 min). Erythrocyte membranes were recovered from the supernatant, and this cell fraction was submitted to repeat steps of ultra centrifugations (100 000 × g for 1 h at 4°C) followed by washing until iRBC ghost were colourless, and stored at -80°C. The same protocol was used to collect membrane proteins from uninfected erythrocytes. Membrane protein extracts were then suspended in 4% (w/v) CHAPS (Sigma). Membrane aggregates were then disrupted by ultrasonication (Vibracell 72412, Bioblock Scientific, Illkirch, France) for 5 min on ice at maximum amplitude and precipitated in 100% acetone (Sigma) to discard lipids. Protein concentration was estimated using Lowry-based DC assay (Biorad, Hercules, CA, USA) according to the manufacturer's instruction. Membrane protein extracts were suspended in a buffer containing 8 M urea (Sigma), 2 M thiourea (Sigma), 4% (w/v) CHAPS (Sigma) and 30 mM Tris (Sigma), adjusted to pH 8.5 in order to obtain a protein concentration adjusted to 2.5 μg/μL.

### Immunoblots and analysis procedures

The RBC and iRBC membrane samples were reduced in a Tris buffer containing dithiothreitol (1% w/v, Sigma), and 15 μg of each sample were loaded per well onto a 10% polyacrylamide gel before to be separated by SDS-PAGE in a Mini PROTEAN II (BioRad, Hercules, CA, USA). After SDS-PAGE, gels were transferred onto a nitrocellulose membrane (0.45-μm, GE Healthcare) by semidry blotting [29]. Each membrane was cut into 18-22 strips (3-4 mm wide), before incubation with a saturation buffer (5% non-fat dried milk in PBS containing tween-20 (0.1% v/v, Sigma)). Immunoblots were carried out with human sera, which were diluted at 1/100 in saturation buffer. After overnight incubation, blots were incubated with mouse anti-human Fcg/IgG horse radish peroxidase (HRP) conjugated antibody (1/5 000, Beckman Coulter, San Jose, CA, USA), and revealed using an ECL Plus western blotting (WB) detection system (GE Healthcare). Densitometric analysis of autoradiographs was performed using Diversity database™2.2 software (Biorad) to align and compare the IgG immune patterns. Two sera, one from a NEI and another one from a HEI which were used as controls, were tested on each blot in order to assess the quality of each immunoblot and to improve the accuracy of the alignment of antibody reactivities intra- and inter-gels [30,31]. Molecular weights were estimated by comparison with standard molecular weight (Biorad).

### CyDye labelling

Membrane proteins from iRBC were minimally labelled with CyDye according to the manufacturer's recommended protocols (GE Healthcare). Briefly, membrane protein extracts (50 μg) were labelled with 400 pmol of Cy5, freshly dissolved in anhydrous dimethyl formamide (Sigma), and incubated on ice for 30 min in the dark. The reaction was quenched with 1 μL of free lysine (10 nM, Sigma) by incubation 10 min on ice. An equal volume of 2× sample buffer (8 M urea, 2 M thiourea, 4% (w/v) CHAPS, 10 mM DTT, 1% (v/v) IPG Buffer 3-10 (GE Healthcare) was added to the Cy5 labelled sample before submission to two-dimensional electrophoresis (2-DE).

### Two-dimensional electrophoresis (2-DE)

Reagents that were not purchased from GE Healthcare, are indicated. Destreak buffer containing 1% (v/v) IPG buffer 3-10 was used for IPG strips (18-cm) overnight rehydratation. The samples were laid at the acidic end of the IPG strip using a cup-loading technique. IEF was carried out on an Ettan IPGphor II electrophoresis unit at 20°C, for a total of 45 kVh. Prior to separation in the second dimension, IPG strips were reduced and alkylated in a equilibration buffer containing 50 mM Tris-HCl, pH 8.6 buffer, 6 M urea, 2% SDS and 30% glycerol supplemented with 1% (w/v) DTT or 2.5% (w/v) iodoacetamide instead of DTT for 10 min. IPG strips were then placed on the top of 10% uniform polyacrylamide gels. Strips were overlaid with 0.5% agarose in 1× running buffer containing bromophenol blue and the proteins were further separated by SDS-PAGE (15 W per gel) at 20°C, in Ettan DALT Six electrophoresis system. Two-dimensional gels were either stained with Imperial™Protein Stain (Thermo Scientific, Rockford, IL, USA) or transferred onto nitrocellulose membranes and incubated with human sera as described above (see Materials and Methods section: immunoblots and analysis procedures). For nitrocellulose membranes with Cy5-labelled proteins, antibody response was revealed using goat-anti-human IgG FITC-conjugate (diluted at 1:2000) (Invitrogen). Immunoblots were directly digitalized using a Typhoon™Trio Image scanner. Images were analysed with Decyder v6.5 software, allowing an accurate spot matching between 2-D protein patterns and 2-D antigenic patterns from gels and immunoblots respectively. Matched spots were selected for excision and further identification by mass spectrometry (MS).

### Spots excision and in-gel digestion

Based on Decyder analysis, spots of interest were excized using Shimadzu Biotech Xcise System (Champs sur Marne, France). Protein spots were digested overnight at 37°C with sequencing-grade trypsin (12.5 μg/mL; Promega Madison, WI, USA) in 50 mM NH_4_HCO_3 _(Sigma). The resulting peptides were extracted with 25 mM NH_4_HCO_3 _for 15 min, dehydrated with acetonitrile (ACN) (Sigma), incubated with 5% acid formic (Sigma) for 15 min under agitation, then dehydrated with ACN, and finally completely dried using a SpeedVac. Samples were then stored at -20°C before analysis by mass spectrometry (MS).

### Mass spectrometry analysis

The samples were analysed on a LCQ DecaXPplus (ThermoFinnigan, San Jose, CA) ion trap. Nano-liquid separation of peptides was carried out using an Ettan MDLC chromatographic system (GE Healthcare) in high throughput configuration. Ten microliters of the digest were first trapped on a zorbax 300SB-C18 5 × 0.3 mm column and eluted at a flow rate of approximately 200 nL/min on a zorbax 300SB-C18, 3.5 *μ*m, 150 × 0.075 mm by a linear gradient of eluant B (0.1% Formic acid, 84% ACN) in eluant A (1% Formic acid). Chromatographic system was piloted by Unicorn 5.01 software (GE Healthcare). MS measurements were performed using a LCQTM Deca XP Plus ion trap mass spectrometer (ThermoFinnigan) equipped with a LCQTM nanospray ionization source. A spray voltage of 1.8 kV was applied to the liquid junction via an in-union high voltage contact coupled to a silicaTip emitter (New Objective). Operation of the mass spectrometer was fully automated during the entire procedure using Excalibur 1.3 data system (ThermoFinnigan). Continuous cycles of one full scan (m/z 500 to 1700) followed by three data-dependent MS/MS measurements at 35% normalized collision energy were performed. MS/MS measurements were allowed for the three most intense precursor ions with a maximum rejection time limit of 1 min. All MS/MS spectra were sequence database searched using Mascot Daemon v2.2.2 software (Matrix Science, London, UK).

### MS data analysis

The data were searched using Mascot software, against *Homo sapiens *and *P. falciparum *National Center for Biotechnology Information non-redundant (NCBInr, NIH, Bethesda, MD) protein database (June 10^th^, 2009). Search parameters are set in order to allowed for one missed tryptic cleavage site, the carbamidomethylation of cysteine, and the possible oxidation of methionine; precursor and product ion mass error tolerance was <0.2 Da. All identified proteins have a Mascot score greater than 41 (Mixed: *H. sapiens + P. falciparum*, 237,583 sequences), corresponding to a statistically significant (*p *< 0.05) confident identification. Moreover, among the positive matches, only protein identifications based on at least two different non-overlapping peptide sequences with a mass tolerance <0.05 Da were accepted. For single peptide-based identification, in addition to Mascot score significance, were considered only peptide sequence with at least six consecutive amino acids detected on MS spectra (Additional file 1). These validation criteria were added in order to limit the number of false positive matches without missing real proteins of interest.

### Statistical analysis

For IgG antibody patterns analysis, the data were expressed in binary mode (0, absence of antigenic band; 1, presence of an antigenic band). The Mann-Whitney and the Fisher exact test were used as appropriate. All statistical analyses were done with the SPSS 12.0 software.

## Results

### BEI sera selection according to their IgG reactivity against iRBC surface proteins

**Figure 1:**
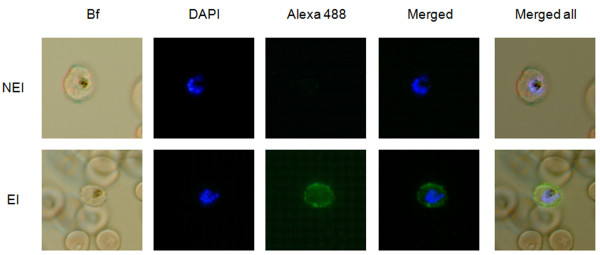
**Evaluation of human serological response against RBC infected by RP8 *P. falciparum *strain by liquid indirect immunofluorescence assay (L-IFA)**. Representative IgG response against iRBC from a non-exposed individual (NEI, upper row) and a briefly exposed individual (BEI, lower row) to malaria, are shown. Specific antibody response to surface proteins from live iRBC was revealed with alexa 488 goat anti-human IgG antibodies (green). *P. falciparum *DNA was stained with DAPI (blue), and merged images with bright field (BF) are shown (Magnification × 100).

IgG antibody reactivity from 751 BEI sera sampled after a five months journey in endemic area was assessed by liquid-indirect immunofluoresence assay (L-IFA). Considering reactions with titres over 1:80 as positive, 38 BEI sera (5.0%) shown an IgG reactivity directed against the surface of iRBC (Figure 1). Among the 38 selected BEI, 37 were males with a mean age ± SD of 25 ± 3.5 years. Eight BEI had a *P. falciparum *clinical episode during the journey. These positive BEI sera were selected for 1 D immunoblotting analysis. Sera from individuals never exposed to malaria (NEI, *n *= *31*) were also tested by L-IFA, and none of them presented IgG reactivity against iRBC at 1:40 dilution (Figure 1). Sera from highly exposed individuals (HEI, *n *= *14*) presented a high IgG reactivity against the surface of iRBC until dilution to 1:320.

### Comparative IgG responses against RBC and iRBC membrane protein extracts between NEI, BEI and HEI

**Figure 2:**
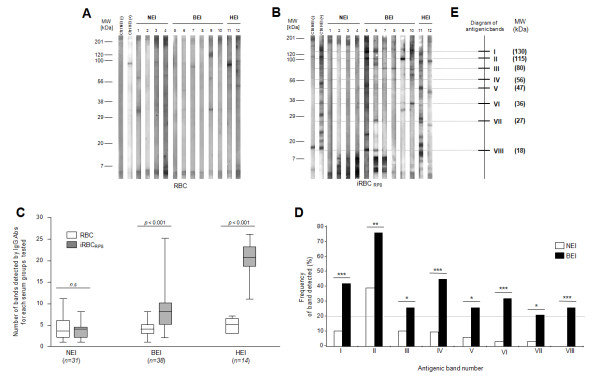
**Analysis of immune response against iRBC membrane protein extracts using sera from non-exposed individuals (NEI), briefly exposed individuals (BEI) or highly exposed individuals (HEI) to malaria**. IgG immune profiles from 4 NEI, 6 BEI and 2 HEI revealed by immunoblotting against erythrocyte membrane protein extracts from RBC (A), or iRBC (B) are presented. Two human sera, "Ctrl NEI (-)" and "Ctrl HEI (+)", loaded on each blot, were used as controls for antibody revelation and migration quality. MW: molecular weight, kDa: kiloDalton. (C) Comparative analysis of the antigenic bands number recognized by sera from NEI, BEI and HEI against RBC and iRBC membrane protein extracts. For this quantitative analysis, significant differences are indicated for each group tested between the two membrane protein extracts (*p *< 0.05, Mann-Whitney test). The line within the box represents the mean number of antigenic bands recognized by each sera group. The boundaries of the box indicate the 25th and 75th percentiles. Whiskers above and below the box indicate the maximum and minimum values. (D) Frequency of iRBC membrane antigenic protein bands detected by NEI and BEI. Analysis of IgG profiles using diversity database software (Biorad) allowed us to exhibit 8 antigenic bands from iRBC membrane protein extracts statistically significant (Fisher exact test, *p < 0.05; **p < 0.01, ***p < 0.001) between NEI and BEI groups. Only protein bands significantly different between NEI and BEI and detected by at least 20 percent of the individuals from a group were indicated. (E) These antigenic bands are indicated on a diagram using Roman numeral numbers, and their corresponding molecular weights are indicated in brackets.

The IgG antibody response of selected sera from NEI (*n *= *31*), BEI (*n *= *38*) and HEI (*n *= *14*), was tested successively against RBC and iRBC membrane protein extracts using immunoblots (Figure 2A &2B). The densitometric analysis of the different patterns of proteins recognized by IgG obtained with regard to the molecular mass, were performed for quantitative comparisons. Against RBC membrane protein extracts, 3.4 ± 2.6 (means ± SD), 3.5 ± 2.1 and 4.9 ± 1.5 antigenic bands were detected per strip for NEI, BEI and HEI sera, respectively (Figure 2C). The number of antigenic bands recognized by each sera group, against RBC membrane protein extracts, was low and no significant difference was observed between these groups (Mann-Whitney test, *p *> 0.05). Against iRBC membrane protein extracts, 3.4 ± 2.0 (means ± SD), 8.1 ± 4.4, 19.9 ± 4.0 antigenic bands were detected per strip for NEI, BEI and HEI sera, respectively (Figure 2C). In this case, the number of antigenic bands recognized by BEI and HEI groups were significantly more important compared to NEI group (Mann-Whitney test, *p *= 1.6 × 10^-7 ^and *p *= 4.5 × 10^-7 ^respectively). Moreover, the number of antigenic bands detected between RBC and iRBC membrane protein extracts is significantly increased for BEI (Mann-Whitney test, *p *= 8.2 × 10^-8^) and HEI (Mann-Whitney test, *p *= 4.9 × 10^-8^). For NEI group, between RBC and iRBC membrane protein extracts, the number of antigenic bands detected was not significantly changed (Mann-Whitney test, *p *> 0.05). Collectively, these results suggest that individuals exposed to *P. falciparum *parasites (BEI and HEI) present an IgG immune response specifically directed against iRBC membrane protein extracts. It is interesting to note that no antigenic bands could be detected above 200 kDa.

### Determination of antigenic bands recognized by BEI sera

In order to define band identity of antigens recognized by BEI, a qualitative analysis of the IgG immune response was performed between NEI and BEI groups. A mapping and alignment of the IgG patterns allowed us to detect a total of 23 and 36 distinct antigenic bands against RBC and iRBC membrane protein extracts, respectively. Against RBC membrane protein extracts, no antigenic band was significantly detected more frequently in one group than to the other one (Fisher exact test, *p *> 0.05). Conversely, against iRBC membrane protein extracts, eight antigenic bands were significantly detected more frequently in BEI group compared to NEI group (Fisher exact test, *p *< 0.05; Figure 2D). Each of these antigenic bands was detected by at least 20% of BEI (Figure 2D). Additionally, each BEI sera recognized at least two of the eight selected antigenic bands. The molecular weights of these antigenic bands ranged from 18 kDa to 130 kDa, and a schematic representation of these antigenic bands was depicted on an immunoreactivity diagram (Figure 2E). Among the BEI group, five sera were selected with regard to their IgG response on 1-D immunoblots for subsequent antigens identification. Each selected BEI sera recognized at least three of the eight discriminatory antigenic bands and the pool covered the whole spectrum of these antigenic bands against iRBC membrane protein extracts. The pool was composed of these five selected BEI sera.

### Characterization of iRBC membrane antigens recognized by BEI

**Figure 3:**
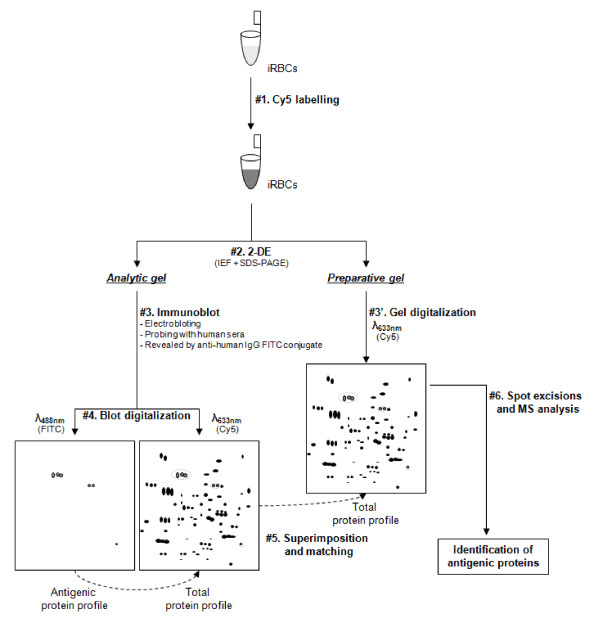
**Schematic representation of experimental workflow for an accurate identification of antigenic protein using 2-D immunoblot with a fluorescence-based method**. The different steps are numbered from #1 to #6. Briefly, iRBC membrane protein extracts were pre-labelled with Cy5 cyanine (#1), separated by 2-D electrophoresis (#2), electroblotted onto membranes, probed with the pool from 5 selected BEI sera and incubated with a goat-anti-human IgG FITC-conjugate (#3). Following blot digitalization at Cy5 and FITC wavelengths (#4), two images corresponding respectively to the antigenic protein pattern and the total protein expression profile were obtained. The superimposition of these two images (#5) allowed us to excize accurately spots of interest on preparative gel run in parallel (#3'), before to submit them to mass spectrometry (MS) for identification (#6). Protein expression profile was used as "internal standard" to perform a perfect match between the blot and the preparative gel.

To further characterize these discriminatory antigens, a 2-D immunoproteomic approach was adopted. A major difficulty of this approach is to perform a perfect match between antigenic spots detected on 2-D immunoblot and their counterparts on the preparative gel, on which matched spots will be excized and further analysed by mass spectrometry (MS). As the results of these protein identifications are the starting point of further studies, it is necessary to limit miss matching. Thus, 2-D immunoblot using a fluorescence-based method allows to increase considerably the confidence in spot matching between preparative gel and immunoblots (Figure 3).

**Figure 4:**
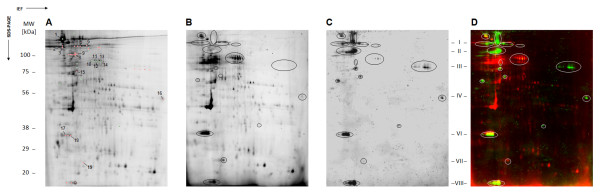
**Determination of protein spots detected by BEI sera on iRBC membrane protein extracts by 2-D immunoblotting**. A 2-D immunoblot using a fluorescence-based method was performed to detect precisely and accurately antigenic protein spots. (A) Protein profile of iRBC membrane protein extracts pre-labelled with Cy5 and separated by 2-D electrophoresis. (B) Protein profile of the same protein sample used in "A" after Cy5 labelling, separation by 2-D electrophoresis and electroblotting onto nitrocellulose membrane. (C) Immunoblot pattern of a pool of 5 selected BEI sera revealed with a goat-anti-human IgG FITC-conjugate against iRBC membrane protein extracts. (D) Merged images of the electrobloted protein profile (*e.i*., "B") and the immunoblot pattern (*e.i*., "C"). Antigenic spots detected onto immunoblot (*e.i*., "C") are ringed and reported onto electrobloted protein profile (*e.i*., "B") and merged images (*e.i*., "D"). Antigenic spots which were excized for mass spectrometry (MS) analysis are marked with red or green circles onto gel image and those which were identified are indicated by Arabic numbers (*e.i*., "A"). Selected antigenic bands are indicated by Roman numbers and positioned according to their MW. Antigenic spots corresponding to selected antigenic bands are indicated by red circles, the others antigenic spots are assigned by green circles (A). A resume of the correspondence between Roman and Arabic numbers is indicated in additional file 2. MW: molecular weight, kDa: kiloDalton.

The pool of BEI sera allowed to detect 42 antigenic spots onto the 2 D immunoblot (Figure 4). To facilitate the alignment of the antigenic bands with the corresponding spots between 1 D and 2 D immunoblot, iRBC membrane protein extracts were loaded onto 2 D gel beside the IPG strip, prior electrophoresis, as described previously [30]. For seven out of eight antigenic bands selected by 1 D analysis, their corresponding spots were detected onto 2 D immunoblot (Figure 4). No antigenic spot corresponding to the molecular weight (MW) of band "V" was detected onto 2 D immunoblot. This could be attributable to the extremes pI or low solubility of this antigenic protein in the buffer for 2DE.

As some bands split into several protein species, 30 spots were assigned to the seven antigenic bands (Figure 4, Additional file 2). A typical example is presented by the antigenic band number "I" which can be resolved into nine different spots on the 2-D immunoblot. The 42 antigenic spots detected onto the immunoblot, were excized and submitted to MS for identification (Figure 4). The resulting fragment ion spectra were searched against *H. sapiens *and *P. falciparum*-protein databases (NCBInr). Nineteen protein spots (45%) were successfully identified and correspond to 13 distinct proteins according to their NCBI accession number (Additional file 2). Among them, four were identified as *P. falciparum *proteins and nine as *H. sapiens *proteins. Selected bands "I", "II" and "VII" were identified as *H. sapiens *antigens and selected bands "III", "IV" and "VI" as *P. falciparum *antigens. However, MS did not identify protein spots corresponding to the selected band "VIII", despite their apparent high abundances. This could be attributable to the low MW of these spots giving spectra with low complexity. Thus, spots corresponding to six out of eight antigenic protein bands were identified.

## Discussion

A recent study performed in the laboratory shown that 35% of soldiers who travelled for a few month period in tropical Africa, presented a serological response against pre-erythrocytic antigens. Among them, only 2% had experienced a clinical malaria attack [22]. Others have reported that travellers from malaria-free countries can develop antibody response against pre-erythrocytic [20,21] and blood stages [32]*P. falciparum *antigens without progressing to a symptomatic illness. Thus, the characterization of erythrocytic stage antigens specifically detected by healthy adults shortly exposed to malaria and under chemoprophylaxis could be informative to estimate individual exposure to malaria transmission, and to clarify the mechanisms involved in the development of immune responses after malaria infection. In this aim, a comparison and an analysis of serological responses between NEI, BEI and HEI against membrane protein extracts from uninfected and *P. falciparum *infected RBC were performed. Although the use of a prophylactic treatment limiting the abundance of the blood malaria parasite could affect the antibody response development, it was observed that 5% of soldiers shown an IgG reactivity directed against the surface of iRBC. Among them, only a minor part (21%) presented a history of clinical malaria. Thus, although exposure to malaria transmission was brief and probably low because of the use of chemoprophylaxis and anti-vector devices, some BEI could develop an antibody response against blood stage antigens without history of clinical malaria attacks.

Analysis of IgG immune response indicated an association between the exposition level and the number of bands revealed by 1-D immunoblotting against iRBC membrane protein extracts. A thorough qualitative analysis of the immune profiles shown that eight protein bands from iRBC membrane protein extracts were significantly more frequently recognized by BEI sera than NEI sera. To identify unambiguously these antigenic bands, an original 2-D immunoproteomic approach using a fluorescence-based method was performed. Almeras *et al *previously demonstrated that it was possible to align, with a good confidence, antigenic bands detected by 1 D immunoblot with the corresponding spots onto 2 D immunoblot [30]. Here, this study shown that a perfect match is now conceivable between antigenic spots from the blot and their corresponding spots from the preparative gel, as recently described by Donoghue *et al *[33]. This method allowed us to identify 13 protein spots corresponding to six out of the eight antigenic bands.

Surprisingly, spots corresponding to bands "I", "II" and "VII" were identified as *H. sapiens *proteins. Auto-antibodies (aabs) against RBC membrane proteins have been already described in malaria patient's sera [34,35]. Little is known about the mechanisms that underlie the aabs production during parasite infection. Aabs production could arise from cross reactivity between host and parasite antigens. This molecular mimicry phenomenon could reduce the tolerance to self antigens and develop an auto-immunity during parasite infection [36]. However, here, all discriminatory antigenic proteins were exclusively recognized on iRBC membrane protein extracts (Additional file 3). The aabs emergence against host proteins on iRBC membrane protein extracts could be attributed to others phenomenon such as protein post-translational modifications. Effectively, it was demonstrated that protein phosphorylations of host and parasite occur during *P. falciparum *infections [37,38]. Moreover, ankyrin (gi|178646; band "I"), which was identified as a host discriminatory antigenic protein, was reported to be targeted by falcipain-2, a *P. falciparum *protease [39]. This protein cleavage could produce neo-antigens which are then recognized only in infected conditions. Nevertheless, aabs directed against these structural membrane proteins were also observed in other *Plasmodium *species infections [35] and could occur in other diseases involving RBC abnormalities like thalassaemia or autoimmune haemolitic anaemia [40,41]. Despite a significant recognition of these host proteins by BEI sera, these discriminatory proteins did not seem to be relevant markers of malaria exposure. Another explanation of the identification host proteins could be attributed to that the spotted area could contain a mixture of both parasitic and human proteins, and the amount of parasite proteins is under-represented compared to human proteins, and so parasitic antigenic proteins may be missed by MS.

Four *P. falciparum *proteins (exported protein 2 (Exp-2) PF14_0678, band "VI"; early transcribed membrane protein (Etramp5) PFE1590w, band "VI"; Heat shock 70 kDa protein (Pf-Hsp70-1) PF08_0054, band "III"; elongation factor 1α (EF1 α) PF13_0304, band "IV") corresponding to three discriminatory antigenic bands were also identified. Exp-2 and Etramp5 which are iRBC membrane associated proteins, have been described to participate to protein transport between parasite and iRBC [42-44]. An immune response against these proteins was previously observed using sera of travellers and individuals from endemic areas [17,44]. The absence of signal detection by sera from never-exposed individuals suggests that Etramp5 could be an interesting marker of *P. falciparum *exposure.

The *Pf*-Hsp70 was detected at several parasite stages into the human host, in sporozoites [45], in liver stage [46,47], in all parasite blood stages [48,49] or in association with the iRBC plasma membrane [42], and is also possibly present on the merozoite surface [50]. Here, a specific antibody response against *Pf*-Hsp70 was revealed by BEI sera. This protein has been described as a major target in the acquisition of immunity in naturally infected humans living in areas of endemic malaria [17,49,51]. Moreover, *Pf*-Hsp70 is able to elicit protection of *Saimiri sciureus *monkey against the asexual blood stage of *P. falciparum *[52]. Collectively, these data suggest that *Pf*-Hsp70 seems to be an immunodominant antigens following parasite infection, and could be considered as a biomarker of malaria exposure.

The EF1 α is an abundant protein constituting 1-2% of the total protein in eukaryotic cells [53], and is an essential component of the translational machinery. This protein is involved in other processes as protein degradation, signal transduction and in the regulation of cytoskeletal rearrangements [54,55]. EF1 α protein was unambiguously recognized by BEI sera reflecting an exposition to the immune system. It is the first time that EF1 α protein was described as antigenic in individuals exposed to malaria.

The present study failed to identify several well described *P. falciparum *antigenic proteins from the iBRCs plasma membrane. The major part of these *P. falciparum *antigens are large hydrophobic proteins (> 150 kDa) which are generally under represented and can be difficultly detected on 2-D electrophoresis [56], such as PfEMP1 [57], Pf332 [58,59], the cytoadherence linked asexual protein 9 (Clag 9) [60,61] or the erythrocyte-binding antigen 175 (EBA-175) [62]. Furthermore, it was reported that serological immune responses against several *P. falciparum *antigens is conformation dependent [63,64]. Thus, the reducing and denaturing conditions used for the immunoblot analysis in the present study, did not allow to take into consideration conformational epitopes. Conversely, antibody reactivities against linear epitopes derived from PfEMP1 or other *P. falciparum *proteins have been already observed under reducing conditions [65,66]. The non-detection of these large proteins on 1-D and 2-D immunoblotting could be then attributed to incomplete protein transfer onto nitrocellulose membrane as previously described [67]. The use of only one parasite strain with limiting variant antigen repertoire, for BEI sera selection, could also induce a bias for identifying more antigenic membrane parasite proteins. Moreover, Fried *et al. *reported that continuous *in vitro *culture of laboratory isolates could conduct to the loss or truncation of some parasite proteins [68], which could participate to this underestimation of antigenic parasite protein detection. Additionally, a lot of known antigens from the asexual blood stage are proteins from the merozoite surface (eg. MSP1, MSP2 or RESA) [69]. In the sample condition preparations used here, iRBC membrane extracts were exempted of merozoites. Moreover, although some merozoite proteins are described to associate to the iRBC membrane during merozoite invasion (eg RSP2) [70,71], these proteins are largely under-representated on the iRBC membrane fraction. Other iRBC membrane proteins such as RIFIN (30-45 kDa) [72] and STEVOR (30-40 kDa) [73], were reported to be antigenic in individuals which have experienced several malaria infections [74,75]. A short exposure to malaria might not be sufficient to induce an antibody response against these proteins in BEI. However, complementary methods, such as the screening of *P. falciparum *expression library with BEI sera, could be envisaged for the characterization of others antigenic parasite proteins, as described previously [76].

The detection of a specific immune response against iRBC membrane extract could be unexpected using sera from individuals which have a mandatory chemoprophylaxis during their journey. Doxycycline was reported to be partially efficient on liver stages of *P. falciparum *parasites [77], and to alter asexual parasite blood stage at the end of the second erythrocytic cycle [78,79]. Thus, a possible discontinuity in chemotherapy observance would have increased the risk to develop a specific IgG response against blood stage antigens.

## Conclusion

This study provides evidence that some BEI could develop a singular antibody response against blood stage antigens after a short exposure to malaria in endemic area. An original immunoproteomic approach allowed the identification of some discriminatory antigenic bands, which corresponded to *P. falciparum *and *H. sapiens *proteins. These antigens may represent promising erythrocytic biomarkers to estimate individual exposure to malaria transmission, and might help to understand the first stages of the immune responses to primary malaria infection. These antigens could be also useful in the analysis of the host-parasite relationships among travellers, or individuals living in areas where malaria is under elimination.

## List of abbreviations

Aabs: Auto-antibodies; BEI: Briefly exposed individuals; CD36: Cluster of differentiation 36; EF1 α: Elongation factor 1α; Etramp: Early transcribed membrane proteins; Exp-2: Exported protein 2; HEI: Highly exposed individuals; Hsp: Heat shock protein; HUVEC: Human Umbilical Vein Endothelial Cells; ICAM-1: Intercellular adhesion molecule-1; iRBC: Infected red blood cells; MHC: Major histocompatibility complex; NEI: Non exposed individuals; PEXEL: *Plasmodium *export element; PTEX: *Plasmodium falciparum *translocon of exported proteins; TVN: Tubovesicular network; PVM: Parasitophorous vacuole membrane; RBC: Red blood cells.

## Competing interests

The authors declare that they have no competing interests.

## Authors' contributions

Conceived and designed the experiments: AL, FT, RC, FA. Performed the experiments: FA, AL, BS, DC. Analysed the data: FA, AL, PM, RC, BM, FT, LD. Contributed reagents/materials/analysis tools: PM, VC, BM, FP. Wrote the paper: FA, AL, RC. All authors have read and approved the final manuscript.

## Supplementary Material

Additional file 1 **Single-Peptide-Based Protein Identifications**. Single peptide-based identifications of the "proteasome subunit HsN3, gi565651" including the spot number, the monoisotopic mass of neutral peptide, the peptide sequence, the ion score, the expect value and the Mascot score are indicated in the table. The observed masses as well as fragment assignments are graphically presented at the bottom. Click here for file

Additional file 2 **Antigenic iRBC membrane proteins detected by BEI sera**. The proteins were identified by LCQ DecaXPplus mass spectrometer. Band and spot numbers corresponds to numbers indicated in Figure 2 and Figure 4, respectively. The identities of protein spots, their NCBI accession numbers, the theoretical and observed MW values, the *pI *values, as well as the corresponding percentage sequence coverage, the number of peptide sequences, and the Mascot score are listed for MS/MS analysis (Protein scores greater than 41 were considered as significant (p < 0.05)). *As a single-peptide was used for this protein identification, the corresponding MS/MS spectrum was included in the additional file 1. Click here for file

Additional file 3 **Specific antigenic protein profiles recognized by pooled sera from briefly exposed individuals (BEI)**. Two-dimensional immunoblots were performed as described previously [31]. Briefly, RBC or iRBC membrane protein extracts were resolved by IEF on pH range 3-10 linear IPG strips (7 cm, GE Healthcare). Before SDS-PAGE (10%), 10 μg of samples were loaded at the left side of the IPG strip, and gels were electrobloted onto nitrocellulose membrane (GE Healthcare). Five sera from each group (BEI, non-exposed individuals (NEI) and highly exposed individuals (HEI)) were selected according to their representative immune profile on 1 D immunoblot, and were pooled. Each pooled sera were probed onto 2 D immunoblot, and antigenic protein spots were revealed using ECL kit on autoradiography X-ray film (GE Healthcare). Representative 1 D (#) and 2 D antigenic profiles obtained with BEI (A and B), NEI (C), and HEI (D) pooled sera against RBC (A) or iRBC (B, C and D) membrane protein extracts are illustrated. Black circles correspond to antigenic protein spots detected on 2 D immunoblots. Antigenic spots detected by BEI and NEI or BEI and HEI pooled sera on iRBC membrane protein extracts are encircled in dashed line (C and D). Roman numbers correspond to antigenic bands indicated in Figure 2 and additional file 2. Click here for file
