# Supplementary material for: Specific antibody responses against membrane proteins of erythrocytes infected by Plasmodium falciparum of individuals briefly exposed to malaria
Source: Malar J. 2010 Oct 11;9:276. doi: 10.1186/1475-2875-9-276 (PMC2959075; doi:10.1186/1475-2875-9-276)
Supplement: Additional file 1 — Single-Peptide-Based Protein Identifications. Single peptide-based identifications of the "proteasome subunit HsN3, gi565651" including the spot number, the monoisotopic mass of neutral peptide, the peptide sequence, the ion score, the expect value and the Mascot score are indicated in the table. The observed masses as well as fragment assignments are graphically presented at the bottom. [file 1475-2875-9-276-S1.DOC]

Additional file 1: Single-Peptide-Based Protein Identifications

| Spot  number | Protein  name | Accession number | m/z | Peptide | Ion score | Expect | Significance  (Mascot score) |
| --- | --- | --- | --- | --- | --- | --- | --- |
| 19 | proteasome subunit HsN3 [*Homo sapiens*] | gi|565651 | 1547.5023 | TQNPMVTGTSVLGVK | 48 | 0.012 | 63 |
