# Supplementary material for: Specific antibody responses against membrane proteins of erythrocytes infected by Plasmodium falciparum of individuals briefly exposed to malaria
Source: Malar J. 2010 Oct 11;9:276. doi: 10.1186/1475-2875-9-276 (PMC2959075; doi:10.1186/1475-2875-9-276)
Supplement: Additional file 2 — Antigenic iRBC membrane proteins detected by BEI sera. The proteins were identified by LCQ DecaXPplus mass spectrometer. Band and spot numbers corresponds to numbers indicated in Figure 2 and Figure 4, respectively. The identities of protein spots, their NCBI accession numbers, the theoretical and observed MW values, the pI values, as well as the corresponding percentage sequence coverage, the number of peptide sequences, and the Mascot score are listed for MS/MS analysis (Protein scores greater than 41 were considered as significant (p < 0.05)). *As a single-peptide was used for this protein identification, the corresponding MS/MS spectrum was included in the additional file 1. [file 1475-2875-9-276-S2.DOC]

**Additional file 2:** Antigenic iRBC membrane proteins detected by BEI sera.

| **Band number** | **Spot number** | Protein name | **Accession**  **no. (NCBI)** | **Accession**  **no. (Pf-locus)** | **MW** **(kDa)** | | ***p*I**  **value**  **(Theor.)** | **Coverage**  **(%)** | **Number of MS/MS**  **peptide sequences** | **Significance**  **(Mascot score)** |
| --- | --- | --- | --- | --- | --- | --- | --- | --- | --- | --- |
|  |  |  |  |  | **Theor.** | **Observ.** |  |  |  |  |
| **Selected antigenic spots** | | | | | | | | | | |
| **I** | 2 | Ankyrin [Homo sapiens] | gi|178646 | - | 207.14 | 131 | 5.8 | 5 | 7 | 211 |
|  | 3 | beta-spectrin [Homo sapiens] | gi\|338441 | - | 247.03 | 131 | 5.1 | 2 | 4 | 52 |
|  | 4 | erythrocyte ankyrin[Homo sapiens] | gi\|226788 | - | 207.33 | 120 | 5.6 | 11 | 16 | 454 |
|  | 5 | Ankyrin [Homo sapiens] | gi\|178646 | - | 207.14 | 120 | 5.8 | 15 | 24 | 489 |
|  | 6 | Ankyrin [Homo sapiens] | gi\|178646 | - | 207.14 | 121 | 5.8 | 20 | 33 | 778 |
| **II** | 7 | adducin 2 isoform a [Homo sapiens] | gi|9257180 | - | 81.26 | 98 | 5.3 | 13 | 8 | 218 |
|  | 8 | adducin 2 isoform a [Homo sapiens] | gi\|9257192 | - | 81.26 | 95 | 5.7 | 14 | 9 | 245 |
|  | 9 | adducin 2 isoform a [Homo sapiens] | gi\|9257192 | - | 81.26 | 94 | 5.7 | 15 | 10 | 230 |
| **III** | 15 | Heat shock 70 kDa protein [Plasmodium falciparum] | gi|123598 | PF08_0054 | 74.75 | 76 | 5.5 | 18 | 12 | 188 |
| **IV** | 16 | elongation factor 1 alpha [Plasmodium falciparum] | gi|124513850 | PF13_0304 | 49.16 | 55 | 9.1 | 12 | 5 | 115 |
| **VI** | 17 | unnamed protein product/etramp5 [Plasmodium falciparum] | gi|829215 | PFE1590w | 10.80 | 33 | 5.0 | 19 | 3 | 96 |
|  | 18 | Exp-2 [Plasmodium falciparum] | gi\|3021540 | PF14_0678 | 33.16 | 33 | 5.3 | 7 | 4 | 44 |
| **VII** | 19 | proteasome subunit HsN3 [Homo sapiens]* | gi|565651 | - | 29.23 | 24 | 5.7 | 5 | 1 | 63 |
| **Antigenic spots** | | | | | | | | | | |
|  | 1 | **Spectrin alpha erythrocytic 1**[Homo sapiens] | gi|115298659 | - | 281.04 | 145 | 4.9 | 45 | 106 | 2249 |
|  | 10 | erythroid protein 4.1 isoform A [Homo sapiens] | gi|182073 | - | 87.06 | 84 | 5.3 | 17 | 10 | 130 |
|  | 11 | erythroid protein 4.1 isoform A [Homo sapiens] | gi|182073 | - | 87.06 | 84 | 5.3 | 18 | 13 | 101 |
|  | 12 | erythroid protein 4.1 isoform A [Homo sapiens] | gi|182073 | - | 87.06 | 82 | 5.3 | 15 | 9 | 176 |
|  | 13 | erythrocyte membrane protein band 4.1, isoform 3 [Homo sapiens] | gi|4758274 | - | 66.76 | 83 | 6.8 | 24 | 12 | 146 |
|  | 14 | erythrocyte membrane protein band 4.1, isoform 3 [Homo sapiens] | gi|4758274 | - | 66.76 | 82 | 6.8 | 26 | 13 | 179 |
