# Supplementary figures and images for: Specific antibody responses against membrane proteins of erythrocytes infected by Plasmodium falciparum of individuals briefly exposed to malaria
Source: Malar J. 2010 Oct 11;9:276. doi: 10.1186/1475-2875-9-276 (PMC2959075; doi:10.1186/1475-2875-9-276)

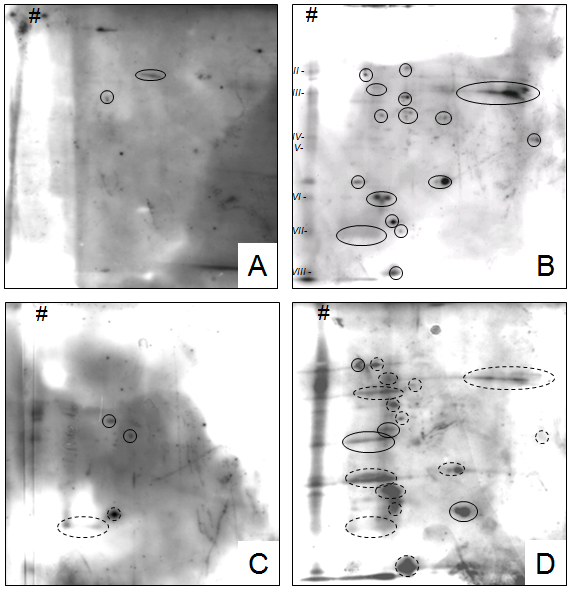

Supplement: Additional file 3 — Specific antigenic protein profiles recognized by pooled sera from briefly exposed individuals (BEI). Two-dimensional immunoblots were performed as described previously [31]. Briefly, RBC or iRBC membrane protein extracts were resolved by IEF on pH range 3-10 linear IPG strips (7 cm, GE Healthcare). Before SDS-PAGE (10%), 10 μg of samples were loaded at the left side of the IPG strip, and gels were electrobloted onto nitrocellulose membrane (GE Healthcare). Five sera from each group (BEI, non-exposed individuals (NEI) and highly exposed individuals (HEI)) were selected according to their representative immune profile on 1 D immunoblot, and were pooled. Each pooled sera were probed onto 2 D immunoblot, and antigenic protein spots were revealed using ECL kit on autoradiography X-ray film (GE Healthcare). Representative 1 D (#) and 2 D antigenic profiles obtained with BEI (A and B), NEI (C), and HEI (D) pooled sera against RBC (A) or iRBC (B, C and D) membrane protein extracts are illustrated. Black circles correspond to antigenic protein spots detected on 2 D immunoblots. Antigenic spots detected by BEI and NEI or BEI and HEI pooled sera on iRBC membrane protein extracts are encircled in dashed line (C and D). Roman numbers correspond to antigenic bands indicated in Figure 2 and additional file 2. [file 1475-2875-9-276-S3.PNG]
